# Neuroprotective Potential of Mild Uncoupling in Mitochondria. Pros and Cons

**DOI:** 10.3390/brainsci11081050

**Published:** 2021-08-08

**Authors:** Dmitry B. Zorov, Nadezda V. Andrianova, Valentina A. Babenko, Irina B. Pevzner, Vasily A. Popkov, Savva D. Zorov, Ljubava D. Zorova, Egor Yu. Plotnikov, Gennady T. Sukhikh, Denis N. Silachev

**Affiliations:** 1 Belozersky Institute of Physico-Chemical Biology, Lomonosov Moscow State University, 119991 Moscow, Russia; andnadya12@yandex.ru (N.V.A.); nucleus-90@yandex.ru (V.A.B.); irinapevzner@mail.ru (I.B.P.); popkov.vas@gmail.com (V.A.P.); zorov@inbox.ru (S.D.Z.); lju_2003@list.ru (L.D.Z.); 2 Kulakov National Medical Research Center of Obstetrics, Gynecology, and Perinatology, 117997 Moscow, Russia; gtsukhikh@mail.ru; 3 Faculty of Bioengineering and Bioinformatics, Lomonosov Moscow State University, 119991 Moscow, Russia

**Keywords:** neuroprotection, brain, mitochondria, uncoupling

## Abstract

There has been an explosion of interest in the use of uncouplers of oxidative phosphorylation in mitochondria in the treatment of several pathologies, including neurological ones. In this review, we analyzed all the mechanisms associated with mitochondrial uncoupling and the metabolic and signaling cascades triggered by uncouplers. We provide a full set of positive and negative effects that should be taken into account when using uncouplers in experiments and clinical practice.

## 1. Introduction

There is a great cluster of knowledge about the protective capabilities of uncoupling of oxidative phosphorylation in the mitochondria of numerous cells, including neural ones, thus compromising chemiosmotic mechanism of energy production [1,2,3,4,5,6,7,8,9,10,11]. According to this mechanism, proton pumps residing in coupling membranes generate the transmembrane potential of hydrogen ions (protons), which is used by ATP synthase to make ATP, thus organizing a tight coupling between the processes of oxidation and phosphorylation. The requisite of this mechanism is a tightly regulated proton cycling existing across the coupling membrane and resulting in a high yield of ATP. The term uncoupling is used concerning the situation when a proton bypasses ATP synthase and electron transport becomes disconnected from the process of ATP synthesis due to a short-circuit of the membrane potential existing across the membrane. It also applies to mitochondria, where uncoupling causes a loss of the tight association of oxidation of respiratory substrates and ATP synthesis [12,13]. Generally speaking, uncouplers exert their action by organizing a proton leak in the inner mitochondrial membrane, which jeopardizes the generation of the proton motive force [14]. Limited usage of uncouplers was announced as one of the most efficient strategies to coupe with different pathologies including aging [15]. In subcellular, cellular and organismal experiments, three uncouplers are the most widely used: DNP (2,4-dinitrophenol; active concentrations are in the region of 100 µM), CCCP (carbonylcyanide-3-chlorophenylhydrazone; active concentrations are about two orders less than of DNP), and FCCP (carbonylcyanide-4-trifluoromethoxyphenylhydrazone; active concentrations are at least one order less than of CCCP).

At the current level of our knowledge on the mechanisms of uncoupling, two modes are distinguished. According to the first mechanism, even uncouplers using a bilayer membrane provide a proton leak, causing a collapse of the proton gradient through the membrane [13,16]. Data in recent years indicate the existence of another mechanism involving special proteins in the coupling membranes that mediate the action of uncouplers [17,18,19,20,21]), while the number of nominal candidate proteins for the role is constantly increasing. It should be noted that there is a fundamental difference between these proteins and natural uncoupling proteins (UCPs) [22], the functioning of which differ from those mentioned.

Meanwhile, the impression arises that moderate uncoupling unambiguously contributes to better resistance to various internal and external challenges, which may attribute this method of manipulating mitochondrial activity to a neuroprotective strategy. However, almost nowhere are the negative aspects considered, which should always be evaluated when using uncouplers in experimental and clinical practice, especially for brain pathologies. Previously, we have already given a similar assessment, weighing all the pros and cons of using mitochondria-targeted antioxidants [23], which we will do in this mini-review discussing beneficial and disadvantageous factors accompanying the application of uncouplers.

## 2. Pros

### 2.1. Anti-Oxygen

The oxygen molecule, even in its triplet state, is a quite strong oxidizer, which, when in the cell, can provide unnecessary oxidation of essential molecules, such as proteins, lipids, and nucleic acids. There is a point of view that the very appearance on Earth of oxygen-utilizing bacteria, and later mitochondria, was evolutionarily dictated by the oxygen menace [24] due to the rise in oxygen in the atmosphere, which was called the Great Oxidation Event or the Great Oxygenation Event [25]. Normally, the supply of oxygen to the cell does not limit the rate of its utilization [26], and the measured values of pO_2_ in the brain do not limit the activity of cytochrome oxidase, which is the main consumer of oxygen in tissue. By definition, uncouplers activate mitochondrial respiration, thereby potentially they reduce the intracellular values of pO_2_. In case it does not reach critical levels when oxygen concentration does not limit respiration, such lowering of intracellular oxygen and thus its toxicity may be considered beneficial.

### 2.2. Anti-ROS

**Figure 1:**
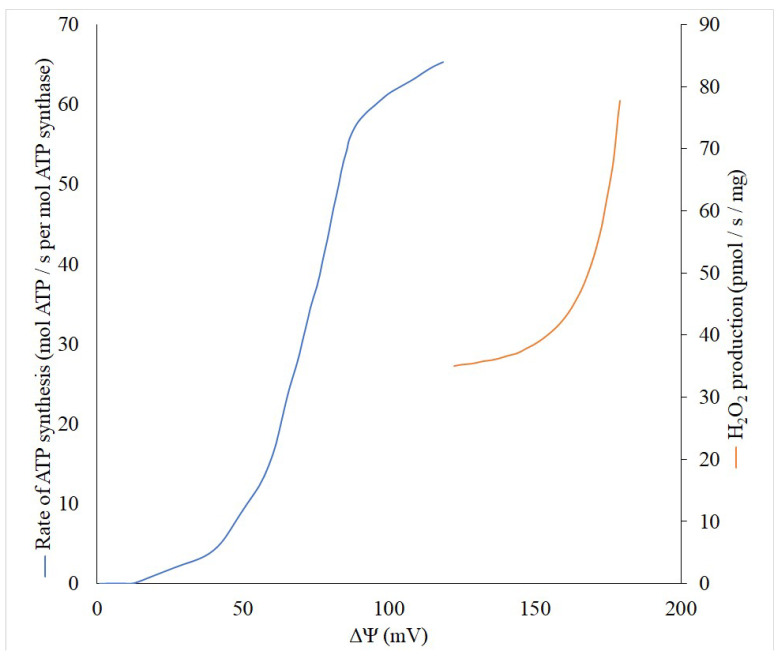
Dependence of the generation of ROS (red) and ATP (blue) on the values of the membrane potential (∆Ψ). ROS generation was measured on isolated rat brain mitochondria with α-ketoglutarate as a substrate (from [30], with modifications). Differences in membrane potential were generated by adding various concentrations of FCCP ranging from 0 to 80 nM. ATP generated was measured using reconstituted E.coli ATP synthase. K^+^/valinomycin diffusion potentials were applied in the presence of ΔpH (from [34] with modifications).

Theoretically, the production of reactive oxygen species (ROS) is a first-order reaction for oxygen, but there are some indicative exceptions, especially in the range of low pO2 values in tissue when an increased generation of ROS is observed [27,28]. However, the generation of ROS non-linearly depends on the membrane potential (Δψ) built in the inner mitochondrial membrane, while reaching an exponential character at high values of Δψ [29,30]. Thus, even a small decrease in the Δψ can lead to a significant reduction in the generation of ROS, and thereby reduce the risk of unnecessary oxidation of important cellular components. This behavior of ROS generation by mitochondria allowed the development of a strategy for combating pathologies accompanied by oxidative stress through using mild uncouplers. These compounds uncouple the processes of mitochondrial electron transport and phosphorylation, but in a very modest fashion, only slightly reducing the membrane potential, ultimately maintaining the ATP at a level that adequately meets metabolic demands. To understand the quantitative relationship between the membrane potential of mitochondria and their ability to generate ATP synthase and produce reactive oxygen species, we performed a literature search and linked these three parameters on one single graph (Figure 1). Although it is impossible to fully match the data available in the literature, first, in both cases, there is a sigmoid dependence of the formation of ATP depending on the membrane potential with the presence of some threshold values, after which, a significant increase in generation begins. Secondly, even in the absence of experimental data for the full range of membrane potentials, it is clearly visible that after 120 mV the ATP generation reaches a plateau. This means that it is possible to reduce the membrane potential without significant violation of the energy balance up to 120–130 mV. Obviously, this can be used to define the concept of “mild” uncouplers, limiting their effective action to the ability to reduce the membrane potential but keeping it not lower than 120 mV. In accordance with modern knowledge, in general, the uncoupling effect of uncouplers which are lipophilic weak acids (p*K*_a_∼4–8), is determined by their ability to be protonated on the side of the membrane where the proton concentration is higher, and translocated to another side. There, after dissociation, the bound proton is released, and the uncouplers return to their original location in the anionic form. The limiting stage of this entire cycle is the transmembrane transport of this anionic form [16]. It is important for uncoupling, which occurs with the participation of fatty acids, for example, caused by derivatives of Skulachev ions having the properties of mild uncouplers [31,32,33]. Thus, the strength (activity) of the uncouplers is partially determined by the rate of transport of the anionic form [16] and uncouplers potentially can be softly divided into two groups: strong and mild (weak), quantitatively discriminated by the active concentrations, from nanomolar to millimolar.

### 2.3. Anti-Obesity

The activation of respiration caused by the use of uncouplers leads to increased mobilization of oxidative substrates and significant activation of oxidative metabolism, while, as the depletion of carbohydrate substrates occurs, the mobilization of fat resources takes place, which is desirable to combat obesity [35,36,37,38]. It should be noted that obesity is one of the risk factors for stroke, including in young adults [31]. Thus, therapeutic uncoupling can have an indirect neuroprotective effect through normalization of metabolism and improvement of the functioning of the cardiovascular system [39].

### 2.4. Increased CO_2_ Production

In parallel with the activation of mitochondrial respiration, there is a proportional increase in the formation of CO_2_, which, in addition to being one of the main factors in maintaining intracellular pH homeostasis, regulates several integral intracellular processes [20,40,41,42,43,44]. Carbon dioxide exerts a direct neuroprotective effect since mild hypercapnia during hypoxia–ischemia provides a long-lasting motor function, as well as neurologic protection for immature brains [45] or traumatic brain injury and stroke (see for review [46]) possibly through increasing cerebral blood flow during hypoxia. Bicarbonate transporters in neural cells were also shown to be protective against ischemia [47,48,49].

### 2.5. Increased Mitophagic Activity

It has been shown that pharmacologic uncoupling or increased contribution of intrinsic uncoupling proteins, including UCPs, lead to the activation of auto/mitophagy (e.g., see [50,51,52]) which is now considered as a positive factor, given the determining role of these processes in the removal of damaged and harmful components of the cell. However, the ambiguity of the data obtained forced us to place the discussion of this issue in the Cons section, where some details of the discrepancies are considered.

### 2.6. Increased Mitochondrial Biogenesis

Uncouplers were found to significantly activate mitochondrial proliferation (biogenesis) [53,54], which is beneficial due to onset of compensatory mechanism designed to preserve mitochondrial ATP production under conditions of toxic mitochondrial damage. In experimental practice, mitochondrial biogenesis is most often associated with the activity of PGC-1α (transcriptional coactivator peroxisome proliferator activated receptor γ coactivator 1α [55], and usually, the proliferative mitochondrial activity is judged by the level of PGC-1α in the cell, which increases after the action of uncouplers. However, in adipocytes, mild mitochondrial uncoupling with FCCP did not stimulate mitochondrial biogenesis [56] which, firstly, raises the question of the lack of universality of the above-mentioned association and, secondly, may discriminate the process of powerful and moderate (mild) uncoupling, which is a function of a dose and chemical nature of an uncoupler. Moreover, while the uncoupler stimulated mitochondrial biogenesis in the oocytes of young animals, this did not happen in old animals [57], which points to another limiting factor that should be taken into account when evaluating the association of uncoupling and mitochondrial proliferation.

### 2.7. Enhanced AMPK Signaling

Uncouplers cause a drop in intracellular ATP through activation of mitochondrial ATPase. AMP kinase (AMPK) is a powerful sensor for the fall of intracellular ATP, reacting through the AMP formed as a result of the hydrolysis of ATP and manipulation of adenylate kinase activity by the mobilization of key metabolic processes. AMPK is one of the most powerful cellular regulatory systems that ensures the optimal balance between ATP production and use [58,59]. It regulates a wide spectrum of different metabolic pathways being highly sensitive to energy disbalance, modifying numerous target proteins by phosphorylating them using ATP. Numerous studies have revealed that activation of AMPK plays a protective role in the brain (for review see [60]). The protective potential of AMPK in different phases of ischemia may be different, and correspondingly, the modulation of its activity may cause variable effects. For example, its activation in the acute ischemic phase may be deleterious [61,62], while preliminary activation of AMPK has been proven to promote neurological autophagy and ameliorate ischemic injury [63].

### 2.8. Anti-Inflammatory

A recent finding includes the anti-inflammatory properties of uncouplers, which reduce the production of pro-inflammatory cytokines, which is especially important in the fight against pathologies, such as sepsis [64,65]. Indirect data show the anti-inflammatory action of uncoupling tightly associated with the expression of uncoupling protein in the brain [66]. The mechanism remains to be determined, but preliminary data indicate the activation of a whole series of signaling pro-survival systems, where AMPK plays an important role [67,68].

## 3. Cons

### 3.1. Possibility of Local Hypoxia/Ischemia

As we have indicated, cellular oxygen reserves are quite limited, and normally the values of intracellular pO_2_ are at quite low levels, due to active mitochondrial functioning, namely oxidative phosphorylation [69]. It is respiratory control that is one of the main regulators of oxygen consumption in the mitochondria, while in a fairly wide range of cell activities there is an equilibrium between the energy supply and energy demand of the cell [70,71]. However, with an increase in the load (for example, caused by an intensification of metabolism due to increased muscle activity or hormonal/emotional load) the subsequent disbalance between oxygen supply and utilization may be observed. Of course, it is necessary to take into account the presence of intracellular oxygen reservoirs in the form of different small metalloproteins, globins [72], including myoglobin in muscle tissue [73] and possibly neuroglobins residing in mammalian neurons [74], apparently playing a certain O_2_ buffering role. Of note, although intracellular aquaporins can facilitate transport of gases within a tissue, such as CO_2_, NO, and NH_3_, the transport of O_2_ through these pores cannot be of physiological relevance due to its very low rate [75], which means that the mitochondrial availability of oxygen can be a limiting factor for its utilization, thus using diffusion as the main mechanism to deliver O_2_ from blood capillaries to mitochondria. However, some authors count four different O_2_-binding globins in the human and rodent brain [76], of which function remains controversial in terms of a potential role in facilitating the O_2_ diffusion. It appears to be very attractive to discuss their role in preventing the concentration of available oxygen to limit the rate of electron transfer along the mitochondrial respiratory chain, which is regarded as the main sign of the onset of hypoxia. In this regard, very indicative is the degree of reduction of the terminal component of the mitochondrial respiratory chain, namely, cytochrome oxidase, which is normally within a wide range of the normal physiological states, is in a completely oxidized state. The appearance of even the slightest signs of cytochrome oxidase reduction (measured by spectroscopy [77,78]) indicates hypoxia in the area in which the cytochrome oxidase reduction is recorded [79]. This is especially fraught for organs with a high metabolism, which include the brain, heart, and kidneys. It is known that the brain can tolerate a lack of oxygen supply to tissues for just a few minutes, after which fatal changes occur, leading to massive death of brain tissue. That is why it is necessary to take any way of activating mitochondrial respiration seriously—whether it is caused by physiological stress or pharmacological induction of respiration, in particular when adding an uncoupler of oxidative phosphorylation. It should be taken into account that the uncouplers can provide maximum activation of mitochondrial respiration, which could compromise the supply of oxygen to the cell. This is why it is important not to allow the use such of concentrations of the uncouplers that can cause maximum activation of respiration to protect tissue from possible hypoxia.

### 3.2. The Drop of ATP Synthesis

By definition, uncouplers compromise oxidative phosphorylation and can completely cease ATP synthesis [14,80]. In general, the synthesis of ATP is determined by the value of the transmembrane potential of hydrogen ions (ΔµH^+^) [81,82], while the proton gradient on the inner membrane of the mitochondria is discharged through the rotation of the rotary part of the ATP synthase complex, driven by the membrane potential (Δψ). Uncouplers reduce the membrane potential, thereby inhibiting the ATP synthetic activity of mitochondria, the termination of the functioning of which can lead to an energy crisis in the cell and a possible switch to less efficient glycolysis in terms of ATP synthesis. To avoid the onset of such an induced energy crisis, it is necessary to very gently regulate the level of uncoupling with the prevention of the use of those concentrations of uncouplers that will lead to a mismatch of energy needs and the level of ATP generation in the cell.

### 3.3. Acidic Shift

Uncouplers activate ATP hydrolysis (ATPase activity, [83]) leading to, not only a very undesirable decrease in the level of ATP in the cell, but also to corresponding acidification of the intracellular space since ATPase activity is accompanied by proton generation [84]. Acidification can cause unwanted activation of degradative systems, such as proteases, lipases, and nucleases [85,86,87].

### 3.4. The Drop of the Membrane Potential-Dependent Reactions

Uncoupler-induced lowering of the mitochondrial membrane potential retards all reactions driven by the membrane potential, of which there are many [88]. One of the most discussed functions (which is attributed to almost the main function that requires membrane potential homeostasis) is the directed transport of proteins into the mitochondria, without which the existence of the mitochondria itself is impossible, given that the mitochondrial genome provides only a small part of its protein needs [89,90,91,92].

The function of ensuring mitochondrial quality control is directly related to this mechanism, which includes the step of transport into the mitochondria of elements that control the quality of mitochondria [93] and the degradation of poorly functioning or non-functioning mitochondria to preserve a young and healthy phenotype [94,95].

A separate function that depends on the values of the membrane potential is the transport of ions into the mitochondria. Thermodynamically, the direction of the membrane potential ensures the transport of cations into the matrix and the exit of anions from it. Special importance is attached to the electrogenic transport of calcium ions in the mitochondria, the physiological significance of which is very high [96,97,98,99]. However, there are data that Ca^2+^ overload and subsequent cell deterioration may be ameliorated by the use of uncouplers, which reduce the membrane potential-driven inward Ca^2+^ transport in mitochondria [100,101]. Indeed, lowering mitochondrial membrane potential by uncoupling agents yielded a higher level of cell tolerance to cytosolic Ca^2+^ overload caused by the neurotoxic effect of glutamate [102,103].

### 3.5. Diminished Mitophagic Activity?

In recent years, the process of programmed micro and macro destruction of cellular elements (micro and macroautophagy) has begun to attract much attention, given the widespread opinion that a violation of this process will lead to the preservation of damaged structures in the cell and, as a result, to the appearance of a pathological (senile) phenotype (reviewed in [104,105]). This is especially important in relation to the removal of damaged and poorly functioning mitochondria, thereby enabling the preservation of a healthy mitochondrial and cellular phenotype [106,107]. The key role in the process of mitophagy is played by the membrane potential on the inner mitochondrial membrane, as a fundamental factor of the mitochondrial quality control machinery.

Although, as we have said, a large number of scientific teams are involved in studies of mitophagy over the world, some purely energetic elements of the process remain logically and actually unsupported and underexplored, and this primarily concerns the role of the mitochondrial membrane potential. The general statement about the mandatory presence of the mitochondrial membrane potential still stays. However, it is only qualitative in nature, while quantitative estimates of the necessary values of the membrane potential for mitophagy are practically absent. This differs the mitophagy process from ATP or peroxide generation presented in Figure 1, where the threshold values, at which the processes practically do not occur, are obvious.

In fact, this is explained by the very principle of the mitochondrial quality control machinery, where the key factor is the transmembrane potential-mediated transfer of special proteins from the cytosol to the matrix, which are the targets of recycling systems. As a result, in the presence of a membrane potential, one such protein (PINK1) is quickly transported to the matrix and becomes inaccessible to detecting systems, while in the absence of a potential, it anchors on the mitochondrial membrane and becomes the subject of ubiquitylation with subsequent degradation of the entire mitochondrion [93]. However, if we take into account the available data on the role of the membrane potential in the transport of proteins to the mitochondria, in accordance with this principle, only mitochondria completely devoid of potential are subject to labeling and subsequent disposal while slightly damaged mitochondria stay in cellular population. Additionally, in this context, it was extremely puzzling to find an increase in mitophagy after the addition of uncouplers [108]. However, a detailed analysis confirmed that the membrane potential had nothing to do with it, and the regulation comes from a change in intracellular pH caused by uncoupler, since the positive effect of the uncoupler (CCCP) was cancelled by the addition of nigericin which converts ∆pH into ∆Ψ [108]. This shows only one side of the complexity of the process of regulating the mitochondrial quality control process with the participation of mitophagy, when the theoretically necessary participation in the process of the membrane potential can be overruled by other factors, which does not allow the membrane potential to be attributed to the critical regulators of mitophagy. On the other hand, an important element of the mitophagic cascade is the transport of proteins into the mitochondria, which is executed by the TIM22 and TIM23 complexes residing in the inner mitochondrial membrane (reviewed in [109]), but it has been shown that, firstly, 30 to 50% of all proteins transported to the mitochondria do not require a membrane potential (i.e., they do not carry a positively charged cleavable presequences [110,111]), secondly, the transport of those proteins that still continue with participation of the membrane potential depends, almost linearly, on the value of the membrane potential, so mitochondria can afford even some transport at its minimum values [112], and thirdly, the role of the membrane potential may be highly specific for different transported proteins requiring a high potential for the transport of some proteins, while for others it is sufficient to have a small potential, depending on to which compartment (external or internal membrane or matrix) this protein is targeted ([113], reviewed in [114]). Except hypothetical provision of electrophoretic transport driven by the membrane potential, the latter causes dimerization of TIM23 which is a requisite for a matrix-targeted signal sequence binding to TIM23 [115].

The set of conflicting data gives us the only option to make a soft statement that the mitochondrial membrane potential plays a role in mitophagy, preferably stimulating it when the potential is collapsed, thus stimulating an onset of utilization of the process in fully dysfunctional mitochondria. For low-functional mitochondria preserving some membrane potential, additional factors are in play.

### 3.6. Hyperactivation of Oxidative Metabolism, Loss of Cellular Reserves

Again, we must be aware that the metabolism of the brain (or heart and kidneys) is already quite high and any additional activation may be undesirable, including the onset of the imbalance between the delivery of resources and energy use. In general, any hyperactivation is not desirable, and its implementation is permitted only in a short-term mode, which is quite difficult to regulate. That is why hypermetabolism caused by hyperthyroidism is the subject for medical treatment when the activity of the key organs, such as the heart and brain are on the edge (note that thyroid hormones are considered as uncouplers [87,116]). Such activation can cause complete depletion of resources which further can cause unwanted cachexia. Additionally, we cannot exclude the danger of the formation of excessive water associated with the activation of metabolism, which must be removed from the area of its formation, that is, from the mitochondria to prevent local or general edema [87].

### 3.7. Reduction of Redox Signaling

The antioxidative function of uncouplers can potentially retard cell signaling that goes with the participation of ROS, including proliferation, differentiation, and other functions. Our analysis shows that a significant part of cellular signaling, especially when it comes to protective signaling, involves ROS [117,118], which makes us take seriously the use of antioxidants or the activation of natural processes designed to reduce the level of oxidants in the cell. ROS homeostasis in the mitochondria, cells, and organs is a prerequisite for the healthy existence of the biological system.

### 3.8. Thermogenesis

Partial or complete loss of oxidative phosphorylation in mitochondria, in particular achieved by the use of uncouplers, leads to the fact that at least part of the free energy stored during the oxidation of substrates is released without coupling with the synthesis of ATP, that is, this energy is dissipated as heat [119]. Thermoregulatory uncoupling in animals adapted to cold was justified and confirmed earlier [120,121,122] as one of the examples of non-shivering thermogenesis. As a result, the thermogenic function of uncouplers can be attributed to several regulatory physiological functions that are implemented if necessary, that is, in conditions of additional heat generation when exposed to low temperatures. Among the possible physiological uncouplers with moderate thermogenic properties, thyroid hormones and free fatty acids were named [123,124,125]. Modern knowledge includes at least two mechanisms by which uncouplers perform their functions in mitochondria, namely through the organization of passive proton leakage through the bilayer membrane [126] and the protein-mediated implementation of uncoupling [19,127,128,129].

Whatever the mechanism, uncouplers in a general sense are thermogenic, causing local mitochondrial release of heat instead of producing ATP. However, hyperthermia belongs to the major risk factors possibly causing irreversible changes in the brain [130,131,132,133], and the only positive effect of hyperthermia can be observed using short-term temperature increases as a stimulus causing a preconditioning effect [134,135,136]. Moreover, hypothermia has protective properties, which imposes requirements, not only to prevent anything that causes an increase in temperature in the brain, but also to use hypothermia in experimental and clinical conditions to prevent the development of neurological damage [137]. A reasonable explanation was given in that hypothermia suppresses mitochondrial activity, in particular, increasing the degree of coupling of oxidation and phosphorylation as the antipode of uncoupling [138].

## 4. Discussion

A large number of experimental and analytical works have been devoted to the problem of the participation of uncoupling of oxidative phosphorylation in various systems providing a protective effect [1,2,3,4,5,6,7,8,9,10,11], including a recent remarkable analysis of the controlling role of uncoupling in physiology and disease [6]. However, the mechanisms of the positive actions of uncouplers operating in a mild mode remain hypothetical, as does the duality of the effects of the use of uncouplers, providing both positive and undesirable consequences. On the one hand, there is a direct line of evidence, which completely boils down to the fact that all positive effects are associated with a guaranteed decrease in the generation of ROS, in particular due to the oxidation of the mitochondrial respiratory chain components, capable of transferring one electron to an oxygen molecule, leading to the formation of O_2_^−^ with the further generation of other ROS [139]. Of course, this model assumes an increase in the respiratory activity of the mitochondria and the activation of the metabolism of the entire organism. That is, in this model, all the positive effects are within the time interval of the uncoupler’s action. Another model claims that the positive action is delayed (as an example, see [140]), and according to this, uncouplers trigger stimulating cascades. If we talk about neurodegenerative diseases, such as multiple sclerosis, Huntington’s disease, Alzheimer disease, Parkinson’s disease, amyotrophic lateral sclerosis, as well as brain trauma, in all these cases, the activation of respiration is not even considered, and the main protective effects are reduced to the synthesis of cAMP stimulated by uncouplers (although still incomprehensible in nature) [141], which triggers the expression of a large number of genes [142], of which the products can afford a neuroprotective effect.

Our analysis, which can converge these two concepts, boils down to the fact that the second proposed mechanism is one of the examples of mitohormesis [143,144], stimulated by uncouplers. In this regard, a two-phase protective echelon can be implemented, which in the first stage, in the acute phase, reduces the high steady-state levels of ROS observed in the mentioned neurodegenerative states, but which can activate metabolism, with the possible short-term onset of near-ischemic states. These are accompanied by a short-term non-lethal increase in the level of ROS, triggering protective neuroprotective cascades, similar to those that occur during ischemic preconditioning [107,118,145,146].

The search for endogenous uncouplers has, so far, ended with the recognition that fatty acids and thyroid hormones can perform this function. This does not exclude mediators of the uncoupling process caused by uncoupling proteins (UCP 1–4, [22]), the full set of functions of which must be clarified, with the exception of UCP1, of which the main thermogenic function cannot be doubted. However, on the one hand, fatty acids can exhibit both direct uncoupling properties [147] or be mediators of transmembrane proteins: UCPs [148], translocator of adenine nucleotides (ANT) [129] and dicarboxylate carrier [18,149]. Other candidates for the role of an uncoupler, thyroxine and triiodothyronine (T3), have not been sufficiently investigated although in the last century there were indications of their uncoupling ability [150,151,152]. Among recent studies, work has demonstrated that T3 activates mitochondrial respiration via increased oxidation of fatty acids, while simultaneously enhancing autophagic flow and, in particular, mitophagy, deserves special attention [153]. The regulation of mitophagy by natural uncouplers (fatty acids and thyroxine) was also confirmed in experiments on cold exposure of animals, as a result of which the activation of mitophagy was observed in brown adipose tissue [154]. Later it was demonstrated that T3 stimulates brown adipose tissue through enhanced mitochondrial biogenesis and MTOR-mediated mitophagy [153].

In addition, we must be aware that, among commonly used drugs, there are few that possess uncoupling ability. Among these drugs are those used in pain medication, aspirin (acetyl salisylic acid), cholesterol lowering Zocor (simvastatin) and diuretic Lasix (furosemide) (reviewed in [155]), which forces us to carefully monitor symptoms when they are used.

Considering all these presented arguments, the extremely high metabolic activity of the brain requires a very careful approach for therapeutically induced manipulations, accompanied by an increase in the metabolism of neural cells, including neurons, astroglia, and endothelium. In general, the prevention of hypermetabolism of the brain is one of the immutable tasks that follows from knowledge of neuropathophysiology [156,157,158,159,160,161]. In general terms, this means that the therapeutic window of influence on brain metabolism is quite narrow, and if it is applied to uncouplers that increase metabolic activity, the therapeutic window of concentrations is also either very narrow or, due to its chemical nature, their uncoupling activity should be relatively small, accompanied by insignificant toxic properties.

In this review, we presented a black-and-white picture of the use of uncouplers of oxidative phosphorylation as potential therapeutic agents, in particular when using them in neurological applications. Taking into account the possible positive effects of uncouplers, which also have critics who deny the validity of certain statements (for example, see [162]), we show the ambiguity of their use, which, in general, have limitations, consisting in very careful choices of doses so that the positive effects do not outweigh the negative ones. However, the search for optimal uncouplers with a sufficiently wide positive concentration window of action continues, both at the level of basic science and the commercial level.
